# Cognitive, Emotional, and Auto-Activation Dimensions of Apathy in Parkinson's Disease

**DOI:** 10.3389/fnbeh.2017.00230

**Published:** 2017-11-21

**Authors:** Jonathan Del-Monte, Sophie Bayard, Pierluigi Graziani, Marie C. Gély-Nargeot

**Affiliations:** ^1^Social Psychology Laboratory EA 849, Aix-Marseille and Nîmes Universities, Nîmes, France; ^2^Epsylon, Laboratory Dynamic of Human Abilities & Health Behaviors, Department of Sport Sciences, Psychology and Medicine, Montpellier University, Montpellier, France

**Keywords:** Parkinson's disease, apathy, cognitive deficits, emotional deficits, auto-activation deficits

## Abstract

Apathy is one of the most frequent non-motor manifestations in Parkinson's disease (PD) that can lead to a whole range of deleterious outcomes. In 2006, Levy and Dubois proposed a model that distinguishes three different apathy aetiologies in PD divided into three subtypes of disrupted processing: “emotional-affective,” “cognitive,” and “auto-activation.” These three dimensions associated with dopamine depletion present in the pathology would lead to the emergence of apathy in PD. The aim of this mini-review was to describe and discuss studies that have explore links between apathy and the three subtypes of disrupted processing proposed by Levy and Dubois (2006) and as well as the links between these dimensions and dopamine depletion in Parkinson's disease. The lack of consensus regarding the emotional-affective correlates of apathy and the lack of evidence supporting the hypothesis of the auto-activation deficit, do not clearly confirm the validity of Levy and Dubois's model. Furthermore, the suggested association between dopaminergic depletion and apathy must also be clarified.

## Introduction

Apathy is one of the most frequent non-motor manifestations in PD with a range between 7 and 70% (Leentjens et al., 2008; Pagonabarraga et al., 2015). Apathy is generally defined as a lack of initiative and effort to perform everyday-life activities. Furthermore, there is a lack of intellectual interest and initiative regarding personal or social issues and an indifference or flattening of affect (Marin, 1991). Diagnostic criteria proposed by Robert et al. (2009), define dimensions (emotional, behavioral, and cognitive) related to the emergence of apathy. For Levy and Dubois (2006), apathy is a quantitative reduction of goal-directed behaviors (GDB) despite the patient's environmental or physical constraints. This syndrome is clearly distinct from other symptoms such as depression (Marin et al., 1994; Andersson et al., 1999; Kuzis et al., 1999; Kirsch-Darrow et al., 2006; Dujardin, 2007; Pedersen et al., 2009; Oguru et al., 2010; Ziropadja et al., 2012) despite a symptomatic overlap (Marin et al., 1993, 1994; Landes et al., 2001; Boyle and Malloy, 2004). Recently, Thobois et al. (2017) have proposed several aetiologies of apathy, anxiety and depression in PD. The authors have emphasized the role of anatomical, metabolic and neurotransmission abnormalities in these three syndromes. The etiology overlaps, in particular limbic loop dysfunction and neurotransmission abnormalities must lead the research to study more precisely the phenomena involved in apathy in PD. In 2006, Levy and Dubois proposed a specific model that distinguishes three different apathy aetiologies in PD divided into three subtypes of disrupted processing: “emotional-affective,” “cognitive,” and “auto-activation.” These three dimensions associated with dopamine depletion present in the pathology would lead to the emergence of apathy in PD. However, the lack of consensus regarding the emotional-affective correlates of apathy and the lack of evidence supporting the hypothesis of the auto-activation deficit, do not clearly confirm the validity of Levy and Dubois's model. Furthermore, the suggested association between dopaminergic depletion and apathy must also be clarified.

The aim of this mini review was to describe and discuss studies that have explore links between apathy and the three subtypes of disrupted processing proposed by Levy and Dubois (2006) and as well as the links between these dimensions and dopamine depletion in PD.

## Selection method

We identified references through searches of PubMed using the terms “Cognitive functions and apathy,” “Emotion recognition and apathy,” “Motor behavior and apathy,” “Dopamine depletion and apathy” and through searching the lists of references of selected papers. We restrict our search to studies in English and we also searched the reference lists within identified studies. We selected papers on the basis of their relevance to the purpose of this mini-review (Tables 1, 2).

**Table 1:** Studies assessing links between cognitive dimension and apathy in PD.

	**Patients (PT)**	**Healthy control (HC)**	**Apathy evaluation**	**Cognitive domains**	**Emotional domains**	**Auto-activation domains**	**Brain areas**	**Conclusions**
**COGNITIVE DIMENSION**								
Varanese et al., 2011	Apathy: *n* = 23 Nonapathy: *n* = 25	–	Apathy evaluation scale	Short term memory / Recall / Recognition / Learning / Working memory / Attention / Speed information processing / Executive functions	–	–	–	Groups differed in recall (*p* < 0.001) and executive tasks (*p* < 0.001) Memory and executive functions performances proved to be predictors of apathy
Bogdanova and Cronin-Golomb, 2013	22	22	Apathy scale	Global cognitive functioning / Verbal fluency / Working memory / Attention / Executive functions / Visuospatial / Reading task / Verbal command	Alexithymia	–	–	**Apathy**: PT = HC Groups differed (PT < HC): Verbal fluency / Visuospatial / Reading task / Verbal command No correlation between apathy and cognitive functions
Robert et al., 2012	45	–	Apathy evaluation scale	Global cognitive functioning / Executive functions / Verbal fluency	–	–	Right inferior frontal gyrus (BA47) Right middle frontal gyrus (BA10) Right cuneus (BA18) Left anterior insula (BA13)	No correlation between apathy and cognitive functions Positive correlation between apathy level and insula, frontal gyrus and cuneus areas metabolism
Reijnders et al., 2010	55	–	Apathy evaluation scale / Neuropsychiatric inventory / Lille apathy rating scale	Global cognitive functioning	–	**–**	**LARS**: Precentral gyrus / inferior parietal gyrus / Precuneus / Posterior cingulate gyrus / Insula / Inferior frontal gyrus / Cingulate gyrus **AES**: Precentral gyrus / Insula / Inferior frontal gyrus / inferior parietal gyrus / posterior cingulate gyrus **NPI**: Precentral gyrus / Insula / Inferior frontal gyrus / Inferior frontal gyrus / inferior parietal gyrus / posterior cingulate gyrus	No correlation between apathy and cognitive functions Negative correlation between apathy scores (LARS / AES / NPI) and gray matter density value
Isella et al., 2002	30	25	Apathy scale Gp1 (*n* = 10) ≤ 14 15 ≤ Gp2 (*n* = 10) ≤ 18 19 ≤ Gp3 (*n* = 10)	Global cognitive functioning / Memory / Visuospatial / Verbal fluency / Executive functions	–	–	Frontotemporal lobe Temporal lobe	Positive correlation between apathy scores and executive functions performances Positive correlation between apathy scores and bilateral temporal atrophy No correlation between apathy scores and Frontotemporal atrophy
**Review**	**Nb. of article**							
Alzahrani and Venneri, 2015	*N* = 15 for cognitive dimension correlates *N* = 7 for brain areas correlates		Apathy evaluation scale / Neuropsychiatric inventory / Lille apathy rating scale	Global cognitive functioning / Executive functions	–	–	Bilateral anterior cingulate Orbitofrontal cortex Frontotemporal lobe Temporal lobe Precentral, frontal, orbito-frontal, fusiform and cingulate gyri Insula Left supplementary motor regions	Correlation between apathy level and cognitive performances in 14 studies Correlation between apathy level and brain areas altered in 6 studies

**Table 2:** Studies assessing links between emotional dimension, auto-activation dimension, dopamine depletion and apathy in PD.

	**PT**	**HC**	**Apathy evaluation**	**Cognitive domains**	**Emotional domains**	**Auto-activation domains**	**Brain areas**	**Conclusions**
**EMOTIONAL-AFFECTIVE DIMENSION**								
Bogdanova and Cronin-Golomb, 2013	22	22	Apathy scale	Global cognitive functioning / Verbal fluency / Working memory / Attention / Executive functions / Visuospatial / Reading task / Verbal command	Alexithymia	–	–	**Alexithymia:** PT > HC **Apathy:** PT = HC Positive correlation between alexithymia Factor 3 and apathy score
Drapier et al., 2008	17	–	Apathy evaluation scale	Executive functions	Facial affect recognition	–	Subthalamic nucleus—Deep brain stimulation (STN-DBS)	No differences on facial recognition performances between pre- and post STN-DBS No correlation between apathy and facial recognition performances in pre- and post STN-DBS
Robert et al., 2014	36	–	Apathy evaluation scale	Global cognitive functioning	Facial emotion recognition	–	Left posterior gyrus	Low negative correlation between apathy scores and facial emotion recognition (sadness and surprise) Positive correlation between apathy score and Left posterior gyrus metabolism
Martínez-Corral et al., 2010	Apathy: *n* = 12 Nonapathy: *n* = 19	16	Clinical interview	Global cognitive functioning	Facial emotion recognition	–	–	Groups differed on FER (fear, sadness and anger): Apathy < Nonapathy = HC
**AUTO-ACTIVATION DIMENSION**								
Louis et al., 2012	Essential T: *n* = 79 PD*: n* = 39 Dystonia: *n* = 20	80	Apathy evaluation scale	Global cognitive functioning / Executive functions	–	–	–	Groups differed on Apathy: ET = PD = Dystonia > HC
Hassan et al., 2014	26		Apathy Scale Gp1 (*n* = 20) > 14 Gp2 (*n* = 16) ≤ 14	–	–	Postural instability and gait initiation	–	Significant association between apathy level and postural instability
**DOPAMINE DEPLETION**								
Funkiewiez et al., 2004	69	–	Unified Parkinson's disease rating scale	Global cognitive functioning / Executive functions / Memory	–	–	Subthalamic nucleus	Groups differed on cognitive function between pre-and post STN-DBS: Attention: pre- > post Initiation: pre- > post Verbal fluency: pre- > post Groups differed on apathy level between pre-and post STN-DBS: pre- < post
Thobois et al., 2010	Apathy: *n* = 34 Nonapathy: *n* = 29	–	Starkstein apathy scale unified Parkinson's disease rating scale	Global cognitive functioning	–		Dopaminergic stimulation of D2/D3 receptors	Apathy reversible with dopamine agonist
Thobois et al., 2013	Dopamine Agonist: *n* = 19 Placebo: *n* = 18	–	Starkstein apathy scale robert inventory	–	–	–	Dopaminergic stimulation of D2/D3 receptors	Significant apathy reduction in DA group compared to placebo group
**Review**	**Nb. of article**							
Castrioto et al., 2014	*N* = 22 for cognitive dimension correlates *N* = 12 for emotional dimension correlates *N* = 5 for apathy correlates		Apathy evaluation scale Starkstein apathy scale unified Parkinson's disease rating scale	Executive functions/Working memory/Verbal fluency/Reaction time/Decision making	Reward sensitivity/facial emotion recognition/Emotion discrimination	**–**	Subthalamic nucleus and Globus pallidus deep brain stimulation Cingulate cortex/Ventral striatum/Angular gyrus/Orbitofrontal cortex/Insular/Temporal gyrus	Increased apathy induced by dopaminergic treatment reduction allowed by STN-DBS Difficult interpretation of results for emotional dimension Strong results between STN-DBS and verbal fluency and executive functions deficits

## Cognitive dimension of apathy in PD

“Cognitive inertia” refers to the reduction of GDB due to decrease of the cognitive functions needed to elaborate the plan of actions. Several executive functions (EF) play a fundamental role in the activation, maintenance and stopping of GDB. EF are classically associated with activation of lateral prefrontal areas, particularly dorsolateral involved in working memory and cognitive flexibility (Goldman-Rakic, 1987; Petrides and Pandya, 1999; Rolls, 2000). However, EF are multi-localized on several brain regions (Cowey and Green, 1996; Vilkki et al., 1996; Andrès and Van der Linden, 1998, 2001) and would rest on a distributed cerebral network that is not limited to the anterior cerebral areas (Collette et al., 2006). For example, cognitive flexibility was associated with bilateral increase of activity in dorsolateral, inferior parietal, occipital and temporal regions (Berman et al., 1995; Nagahama et al., 1996; Ragland et al., 1997). Inhibition process was associated with various regions located in cingulate, prefrontal, parietal and temporal regions (Taylor et al., 1997; Bush et al., 1998; Garavan and Stein, 1999; Chee et al., 2000; Collette et al., 2001).

Several studies have shown an alteration of the cognitive functioning in PD (Kudlicka et al., 2011; Alzahrani and Venneri, 2015; Mak et al., 2015; Veselý and Rektor, 2016). Apathy was significantly associated with cognitive deficits. More specifically, results showed that apathetic patients had a significant decrease in memory, working memory and EF compared to non-apathetic patients (Alzahrani and Venneri, 2015). Varanese et al. (2011) showed that apathy was a predictor of cognitive decline in PD. However, the link between apathy and cognitive abilities is not so obvious. Some research did not find any link between apathy and cognitive impairments in PD (Robert et al., 2012; Bogdanova and Cronin-Golomb, 2013). Bogdanova and Cronin-Golomb (2013) showed that only alexithymia, but not apathy, correlated with neuropsychological performances. These opposite results could be explained by the different methodologies used. Indeed, Collette et al. (2006), noted that measures for executive tasks implicated non-executive processing that influenced patient performances. Functional neuroimaging techniques may be more relevant to identify the specific relationships between behavior and brain activity (Collette et al., 2006). In this sense, many studies suggested that brain areas activations involved in the cognitive dimensions, could play a fundamental role in the development of apathy (Alzahrani and Venneri, 2015). Several studies showed significant correlations between apathy and low gray matter density values in the bilateral precentral, parietal and frontal gyri (Reijnders et al., 2010) and bilateral temporal lobe (Isella et al., 2002). Positron emission tomography studies (Robert et al., 2012) also showed that a high apathy score was correlated with right prefrontal cortex (PFC) activation and with left dorsolateral prefrontal cortex metabolism. Several studies demonstrated that dopamine depletion was correlated with cognitive impairments in PD (Sawamoto et al., 2008; Jokinen et al., 2009; Ekman et al., 2012). Polito et al. (2012) showed that the level of dopamine in caudate nucleus modulated the glucose metabolism in frontostriatal circuits affecting executive function domains. Impairment prefrontal dopamine signals would play in cognitive dysfunctions (Dubois and Pillon, 1995; Aalto et al., 2005; Ekman et al., 2012; Narayanan et al., 2013; Matsumoto, 2015). The blockade of dopamine receptors of cortico-basal ganglia loops induced cognitive dysfunctions in animals' studies (Sawaguchi and Goldman-Rakic, 1994; Landau et al., 2009; Cools, 2011) suggesting the fundamental role of dopamine to cognitive processing in this circuit.

The links between apathy and cognitive disorders would appear to be strongly demonstrated by the literature confirming the first-dimension validity of Levy and Dubois's model in PD.

## Emotional-affective (EA) dimension of apathy in PD

The “EA processing” of apathy is a reduction of GDB due to an impossibility of linking affective and emotional (positive or negative) signals with ongoing and forthcoming behaviors. “EA” processes are essential to give motivational value to behavior. For Levy and Dubois, the lack of emotional attribution leads to apathy, either by reducing the desire to perform actions and/or by reducing of ability to assess the consequences of future actions. Apathy related to disruption of “Emotional-Affective” processing would be associated with a dysfunction or a lesion of orbital/medial PFC and limbic territories of basal ganglia. Emotional processing disorders, associated with apathy in PD, could be confused with mood disorders such as depression or alexithymia. Indeed, depression syndrome alters positive emotional treatment and promotes negative emotional treatment while apathy syndrome reduces positive and negative emotional treatment. Several studies have showed that PD patients were affected by alexithymia, defined as “*the difficulty to identify and describe own feelings and a more general reduced aptitude to deal with emotions*” (Taylor, 2000). Alexithymia affects between 18 and 30% of patients without dementia (Costa et al., 2006, 2007, 2010; Castelli et al., 2014; Goerlich-Dobre et al., 2014; Enrici et al., 2015). In recent literature, Assogna et al. (2016) showed strong links between alexithymia and depression. Out of the ten articles evaluated, nine highlighted the presence of positive correlation. However, depressive symptoms and alexithymia do not overlap completely in PD (Costa et al., 2010) and for Assogna et al. (2016), depression could be considered as predisposing factor for alexithymia. Concerning links between alexithymia and apathy in PD, previous studies have shown that neuroanatomic dysfunctions overlap. Alexithymia was correlated with frontal and anterior cingulate cortex alterations, like apathy (Tekin and Cummings, 2002; Levy and Dubois, 2006). However, few studies have tried to distinguish apathy from alexithymia. Bogdanova and Cronin-Golomb (2013) have used the *Toronto Alexithymia Scale* (TAS-20) to evaluate alexithymia level and the short version of *Apathy Scale* in non-demented PD patient. TAS-20 is composed by three factors: (i) difficulty of identifying feeling and bodily sensation discriminations (F1); (ii) difficulty of describing feelings (F2) and iii) externally oriented thinking (F3). In this study, results showed that apathy score correlated only with TAS-20 F3 score. On the other hand, alexithymia was associated with flexibility performances and visuospatial abilities, while apathy was not correlated with these cognitive tasks. These results could be explained by a potential continuum between apathy and alexithymia involving altered processes related to the progress of the pathology. In the sense, Bogdanova and Cronin-Golomb (2013) showed that apathy was a strong predictor of high levels of alexithymia.

Several studies have also shown that facial expression recognition (FER) was impaired in PD (Sprengelmeyer et al., 2003; Dujardin et al., 2004a,b; Lawrence et al., 2007; Clark et al., 2008; Herrera et al., 2011; Wagenbreth et al., 2016). On the other hand, many researches have demonstrated the strong link between FER performances and dopamine depletion (DD) (Sprengelmeyer et al., 2003; Assogna et al., 2008, 2010; Péron and Dondaine, 2012; Carriere et al., 2014). Sprengelmeyer et al. (2003) have compared FER in PD patients without or with medication and healthy controls (HC). FER was assessed using the Ekman 60 Faces test-recognition of prototypical facial expressions. Results demonstrated that PD patients presented FER impairment compared to HC. Fear, sadness, disgust and anger recognition was altered in PD patients without medication while only fear and anger recognition was impaired in PD patient with medication. PD patients without medication were significantly altered for disgust recognition compared to PD patients with medication. According to the authors, disgust recognition could be associated with DD in ventral striatum and the dopamine replacement therapy could restore the emotion recognition process (Sprengelmeyer et al., 2003). However, the role played by dopamine in the FER must be deepened and confirmed due to some studies that not confirm the links between dopamine and emotion recognition (Enrici et al., 2015). On the other hand, the literature also remains cautious about the links between FER and apathy. Robert et al.'s (2014) study showed that apathy correlated negatively with FER impairments and results showed a low significant relationship between overall emotion (β = -0.18), sadness (β = −0.06) and surprise (β = −0.1). Some years earlier, Martínez-Corral et al. (2010) found that apathy was significantly linked with fear, anger and sadness recognition deficits. Finally, several studies have found no link between apathy and FER deficits in PD (Drapier et al., 2008; Albuquerque et al., 2014). The different results of the studies could be explained by different confounding factors insufficiently controlled, such as depression and/or medication types (Robert et al., 2014). However, FER impairments and apathy would share the same neuroanatomic loop involving the subthalamic nucleus (STN) (Drapier et al., 2008; Le Jeune et al., 2009; Thobois et al., 2017) and dopaminergic depletion in STN was strongly associated with apathy severity (Castrioto et al., 2014). DD could play an important role in motor (motor areas and putamen), cognitive (dorsolateral PFC and dorsal caudate nucleus) and emotional (orbitofrontal cortex, ventral caudate nucleus and anterior cingulate cortex, nucleus accumbens) loops affected in PD (Steeves et al., 2009). Dopamine could play also important role in the reward evaluation of given behavior and in providing its motivational value (Aarts et al., 2012). Aarts et al. (2014) showed that dopaminergic medication effects were correlated with increase in BOLD signal in the ventromedial striatum, leading to decreased reward-processing performance in PD. Reward-processing impairments related with increase of dopamine in ventromedial striatum lead to impulse control disorders in PD (Aarts et al., 2014). In fact, impulse control disorder in PD was associated with younger age, treatment with dopamine agonists, and use of high doses of Levodopa (Voon et al., 2009, 2010, 2011a,b). Hyperdopaminergic syndrome would be characterized by several non-motor symptoms such as sensation and pleasure seeking, euphoric, hyperactivity while hypo-dopaminergic syndrome would be characterized by several non-motor symptoms such as apathy, indifferent, dysphoria, sadness, suicidal attempt (Castrioto et al., 2014). A recent review of Castrioto et al. (2014), concerning deep-brain stimulation of STN (STN-DBS), showed an increase of apathy after STN-DBS induced by dopaminergic treatment reduction allowed by STN-DBS (Funkiewiez et al., 2004; Thobois et al., 2010). Finally, apathy strongly associated with mesolimbic dopaminergic denervation (Thobois et al., 2010), disappeared with the introduction of dopamine agonist (Thobois et al., 2013). Previous studies have shown that facial emotional recognition deficits and apathy could share the same altered meso-cortico-limbic circuit. However, there are conflicting results regarding the role of certain limbic regions, such as ventral striatum, in apathy. Recently, Chung et al. (2016) showed no link between the degree of apathy and striatal dopamine transporter binding defect in PD, whereas previous studies seem to show the opposite (Remy et al., 2005; Thobois et al., 2010; Santangelo et al., 2015). For Chung et al. (2016), apathy could be mostly associated with lesions or dopamine depletion in extra-striatal regions, such as thalamus, rather than striatal dopaminergic deficits in PD.

The lack of consensus regarding the emotional-affective correlates of apathy do not clearly confirm the validity of Levy and Dubois's model. However, studies results are in line with the implication of this dimension in apathy in PD.

## Auto-activation (AA) dimension of apathy in PD

The last dimension “AA” could be characterized by the dissociation between spontaneous and voluntary behavioral production induced by an external element. Levy and Dubois suggested that the destruction of limbic system and associative territories of basal ganglions would prevent amplification and transfer to the PFC of the signal playing a role in selection and validation of thoughts or behaviors. “AA” disruption would not influence the emotional processing of a situation but would limit the association between a reward and a given behavior and thus switch off that behavior's production. In 2012, Louis et al. have compared apathy and depression level in Essential Tremor cases (ET), Dystonia cases, Parkinson' disease cases (PD) and Normal controls. After control of depressive symptoms, results showed that apathy level was higher in ET, Dystonia and PD cases compared to normal controls, with the PD patients having the highest score. However, this study has not shown a link between apathy and motor troubles. A few years later, Hassan et al. (2014) assessed links between apathy level, depression level and postural instability in PD. This study showed that hierarchical regression revealed that only apathy predicted postural instability. For Levy and Dubois “AA” disruption could share the same neuroanatomic loops that motor signs, such as akinesia. Indeed, the motor cortico-striatal circuit would be a closed loop organized between the motor cortex, premotor cortex and supplementary motor areas, putamen, globus pallidus (GP), substantia nigra pars reticulata, STN and thalamus. The motor loop would consist of direct and indirect pathways. The direct pathway would involve projections from the putamen to the internal segment of the GP (GPi) and then the thalamus. The indirect pathways would involve projections from the putamen first to the external segment of the GP (GPe), then the STN, and finally back to the GPi before relaying to the thalamus (Martinu and Monchi, 2012). Motor and cognitive symptoms could appear in parallel with the disruption of normal function of the putamen and caudate nucleus, associated with almost complete DD in the putamen. In PD, nigrostriatal DD would result a net increase in STN and GPi discharge, but a decrease in GPe discharge, creating an imbalance between the direct and indirect pathways (DeLong and Wichmann, 2007). Specifically, the indirect pathway would become hyperactive and the direct pathway would become hypoactive, resulting in an excess of inhibitory output from the GPus, leading to bradykinesia and rigidity (Bergman et al., 1994). Apathy associated with “AA” alteration may result from basal ganglia lesions located in the associative and limbic territories, in particular in the internal segment of the GP. García-Cabezas et al. (2007) showed that limbic territories were influenced by the efferent projections of innervated nuclei depending of thalamic dopamine action. In primate, thalamic dopamine would act on the whole cerebral cortex, specifically on to frontal and limbic areas, motor areas, dorsal striatum and amygdala. Thalamic dopamine could modulate emotion, cognition and motors functions. McFarland and Haber (2000) showed that interconnected ventral thalamic and cortical motor areas projected to the same dorsal striatum region suggesting that both areas modulate motor information integration in the striatum. Ventral thalamic would play a role in the corticobasal ganglia loop by giving positive feedback to the striatum to reinforce this circuits necessary for attaining a selected behavior. Rat model of PD confirms this hypothesis by showing that striatal dopamine depletion is associated with several deficits movements-related motor thalamus neural activity (Bosch-Bouju et al., 2014).

Despite the lack of studies confirming the role played by “AA” disruption in apathy, this hypothesis cannot be rejected in PD. Ventral thalamus activity could be an interesting way to better understand the dimension “AA” of apathy in PD.

## Conclusion

Apathy is a complex phenomenon that could find its etiology in three subtypes of disrupted processing: “cognitive,” “emotional-affective,” and “auto-activation.” Nevertheless, contrary to the strong link between “cognitive” dimension and apathy, the lack of consensus regarding the emotional-affective correlates of apathy and the lack of evidence supporting the hypothesis of the auto-activation deficit, do not confirm the role of these dimensions in the emergence of apathy in PD. However, studies highlighting the role of dopaminergic depletion in motor, cognitive and emotional behavioral inhibition, suggest a strong association between hypo-dopaminergic syndrome and apathy in PD. Future research will have to explore these two-possible aetiologies of apathy, in particular by studying the links between stage of disease, dopamine depletion and level of non-motor symptoms. Finally, the potential confusion between non-motor symptoms such as alexithymia or depression, should lead to better specification of the diagnostic criteria of apathy neurodegenerative diseases and more specifically in PD.

## Author contributions

JD-M was involved in the conception, writing, critical review and approval of the final version to be submitted. SB, PG, and MG-N were involved in critical review and approval of the final version to be submitted.

### Conflict of interest statement

The authors declare that the research was conducted in the absence of any commercial or financial relationships that could be construed as a potential conflict of interest.
